# Comparison of performances of SIREN or MFSRS in stroke risk prediction in an African environment

**DOI:** 10.1186/s12889-024-17886-3

**Published:** 2024-02-05

**Authors:** Ifeoma Uchenna Onwuakagba, Emmanuel Chiebuka Okoye, Favour Chidera Kanu, Chukwuemeka Michael Kalu, Christopher Olusanjo Akosile

**Affiliations:** Department of Medical Rehabilitation, College of Health Sciences, Nnamdi Azikiwe University, Nnewi Campus, Nnewi, Anambra State Nigeria

**Keywords:** Stroke risk factors, MFSRS, SIREN, Comparison, Africa

## Abstract

**Background:**

The Modified Framingham Stroke Risk Score (MFSRS) is a widely utilized stroke risk assessment algorithm usually applied in international comparison. The Stroke Investigative Research and Educational Network (SIREN) is the only known African-specific stroke risk assessment algorithm.

**Aims and objectives:**

To compare stroke risk estimates from the SIREN and the MFSRS in an African community.

**Methods:**

This was a population-based cross-sectional survey involving consecutively recruited 310 consenting adult residents (mean age = 37.21 ± 15.84 years) of a Nigerian community. Risk factors of stroke were assessed among the participants and were utilized in calculating stroke risk estimates on the MFSRS and the SIREN. The obtained data were analyzed using descriptive statistics and the Spearman-rank order correlation test at an alpha level of 0.05.

**Results:**

The percentage stroke risk scores estimated by the SIREN and the MFSRS were 34.5% and 6.79% respectively. The most prevalent risk factors among the participants were hypertriglyceridemia (100.0%), raised waist-hip ratio (50.6%), hypercholesterolemia (45.5), physical inactivity (43.2%), psychological stress (41.3%), and hypertension (37.7%). Only two (hypertriglyceridemia and high blood pressure) out of the six factors considered in the MFSRS were rated among the first 10 most impactful risks by the SIREN. There was a weak correlation between the total scores on the MFSRS and the SIREN (rho = 0.39; *p* < 0.01) suggesting that the two ratings were discordant.

**Conclusion:**

There were disagreements between the risk estimates on the SIREN and MFSRS with SIREN having a higher estimate that corresponded with the literature; this may be suggesting a poorer estimation of stroke risks by the MFSRS in an African environment. There is a need for large African-based quality control studies to determine and address these lapses.

## Introduction

Stroke is the second leading cause of death and the third leading cause of disability-adjusted life years (DALYs) lost worldwide [1, 2]. The incidence and mortality of stroke differ between countries, ethnic groups, races, and geographical regions [3]. Over the past three to four decades, the burden of stroke has reduced in high-income countries (due to improvements in prevention, acute stroke care, and neurorehabilitation) [4, 5] but has increased in low- and middle-income countries [1] with incidence rates in the latter now exceeding those in the former [1, 4, 6, 7]. The World Health Organization (WHO) had earlier estimated that by the year 2030, 80% of all strokes will occur in low-and-middle-income countries such as Nigeria [8]. In Nigeria, several population-based studies point to high incidence (0.6–1.63 per 1000 persons) and prevalence (1.14–14.6 per 1000 persons) rates of stroke in different regions of the country [9–12]. In general, the global number of stroke survivors and the global burden of stroke have increased [6, 13]. This is attributable to population growth, aging, improved stroke care, and increased prevalence of modifiable risk factors of stroke [6, 13].

The risk factors of stroke could be classified into modifiable and non-modifiable risk factors [14, 15]. The modifiable risk factors are dynamic as more and more of these modifiable risk factors emerge as time goes by [16]. Ninety percent of all strokes are caused by modifiable risk factors [17, 18] with hypertension, apolipoprotein, current smoking, poor diet, physical inactivity, abdominal obesity, psychosocial factors, cardiac problems, high alcohol consumption, and diabetes mellitus accounting for 91.5% and 87.1% of the population-attributable risk for ischemic and hemorrhagic stroke in the INTERSTROKE study (conducted in 32 countries including Nigeria) [19]. Hence, the overwhelming majority of strokes can be prevented through awareness, control of blood pressure, and lifestyle changes (healthy diet, physical activity, and smoking cessation) [18]. Consequently, rating and controlling of risk factors of stroke have been an important focus of stroke prevention globally, and have led to the formulation of stroke risk evaluation algorithms [14, 20].

In the literature, there are four available stroke risk evaluation algorithms: the Modified Framingham Stroke Risk Score (MFSRS), the QStroke, the Stroke Riskometer™, and more recently, the Stroke Investigative Research and Educational Network (SIREN) [3, 21]. The MFSRS, the QStroke, and the Stroke Riskometer were developed in high-income countries and were therefore tailor-made to suit individuals in these countries. However, with the nature and strength of stroke risk factors varying across races, ethnic groups, and geographical locations [22, 23], these risk assessment algorithms may not perform equally effectively across geographical locations and races. Due to some subtle evidence about the existing evaluation algorithm not being entirely suitable for African environment, Akpa et al. [3] developed the SIREN, a stroke risk score that was designed to accommodate the major stroke risk factors in African environments [3, 24] with Nigeria being one of the countries involved in the development. In addition to the factors (age, sex, dyslipidaemia, blood pressure, and diabetic status) explored in the MFSRS, the SIREN also included other factors deemed important in the African environments (which include sugar, salt, meat, and leafy vegetable consumption, physical inactivity, stress, waist-hip ratio, history and status of cardiac disease, educational attainment, and income level). Apart from the developers, no study seemed to have utilized the SIREN in Nigeria.

Although an accurate assessment of stroke risk factors in each region and population group of the world is important, the benefits of international or interregional comparison of these risk factors can also not be overemphasized. Understandably, this comparison can easily be engendered by a uniformity of risk assessment tools. Though some shortcomings of the MFSRS have been reported, it is still the most popular, utilized, and well-accepted stroke risk prediction score, thereby making it a suitable candidate for this international comparison [25, 26]. The MFSRS has been reported not to factor in modifiable risk factors indigenous to Africans [22] with no study on its applicability and performance in an African environment being available for reference. There seems to be no Nigerian population-based study that has utilized the MFSRS. However, previous Nigerian studies have revealed that the MFSRS/FSRS can be sensitive in differentiating risk scores between different pathological groups [27, 28]. This study was therefore designed to determine the level of convergence in outcomes between the MFSRS and the SIREN in a community in Nigeria. It was hypothesized that the correlation between the MFSRS and the SIREN will be moderate.

## Materials and methods

### Design, setting, and sampling

The present study was a cross-sectional survey involving adult (18 years and above) residents of Nnewi North Local Government Area, Anambra State. Nnewi is a single-town Local Government Area with four quarters. Two out of the four quarters were randomly selected using the Fisch Bowl technique, which was also used in selecting two communities from each of the two selected quarters. Nnewi is an Igbo-speaking metropolitan, semi-urban, commercial, and agricultural community with an estimated population of 1,050,860 in 2020 [29]. It is the second largest city in the Southern Senatorial Zone of Anambra State with a total land mass of about 1,076.9 square miles (2,789 square kilometres) [29]. An advertisement for the study was made in each community through the community, church, and market leaders. Prospective participants were asked to converge at community town halls, church premises, or markets where those who met the inclusion criteria were consecutively recruited. The participants were well-oriented in time, place, and person, and had no communication impairment nor a history of stroke. A sample size of 276 had a 95% power to detect a moderate change of 0.25 at an alpha level of 0.05. The sample size was calculated using the G*Power 3.10.0 program [30]. The study was approved by the Ethical Review Committee of the Faculty of Health Sciences and Technology, Nnamdi Azikiwe University Nnewi, Anambra State, Nigeria. Verbal or written informed consent was obtained from each participant after the purpose and procedure of the study had been thoroughly explained to them. Data collection lasted for six months from January to June 2022. This manuscript conforms to the STROBE (Strengthening the Reporting of Observational Studies in Epidemiology) reporting guidelines [31].

### Instrument for data collection

#### Modified Framingham Stroke Risk Score (MFSRS)

This was used to determine the risk of stroke of the participants in this study. It is a sex-specific health risk appraisal function for the prediction of the first stroke within 10 years among individuals that are up to 30 years of age [32]. The MFSRS is the most well-known and well-accepted stroke risk score and remains the gold standard for predicting stroke risk [22, 33, 34]. It was developed by Wolfe et al. [35] and was later modified by D’Agostino et al. [32] to account for the use of antihypertensive medication. The Modified Framingham Stroke Risk Score (MFSRS) predicts the risk of stroke using age, high-density lipoprotein level, cholesterol level, systolic blood pressure level, diabetes mellitus, and cigarette smoking statuses which are generally categorised according to sex [36]. A predetermined weights are given to participant according to their age (+ 0 to + 15 for men, and + 0 to + 12 for women), high-density lipoprotein level (-2 to + 2 for both men, and women), cholesterol level (+ 0 to + 4 for men, and + 0 to + 5 for women), systolic blood pressure(-2 to + 5 for men, and -3 to + 12 for women) depending on whether the individual is under treatment or not, diabetic status (+ 0 to + 3 for men, and + 0 to + 4 for women) and smoking status (+ 0 to + 4 for men, and + 0 to + 3 for women). The total risk point of an individual is then used to determine their 10-year cardiovascular risk (which can range from < 1.0% to > 30% for both sexes) using predetermined percentage weights. The cardiovascular risk of an individual can then be categorised as low (< 10%), intermediate (10–19%), and high (≥ 20%).

#### Stroke Investigative Research and Educational Network (SIREN)

The SIREN was also used to determine the risk of stroke of the participants in this study. It is a stroke risk assessment that estimates the aggregate stroke risk for indigenous Africans [3]. It was developed by Akpa et al. [3] using a case–control methodology among 3553 pairs of stroke cases and stroke-free controls. They identified 13 potentially modifiable (income level < $100, educational attainment, hypertension, dyslipidemia, diabetes mellitus, cardiac disease, raised waist-hip ratio, stress, sprinkling salt on food at the table, low-consumption of green leafy vegetables, regular sugar consumption, physical inactivity, and regular meat consumption) and two non-modifiable (advancing age and family history of CVD) risk factors associated with the occurrence of stroke. All the predictive variables are dichotomized as either present or absent for each participant. The range of possible total raw scores for an individual by the SIREN scoring system is 0–104 or 0–86 using either the constant weighting or standardized weighting approach respectively (and 0–100 for the transformed score). The Cohen’s kappa for validity was maximal at a total risk score of 56% for both the constant and the standardized weighting methods. The scoring system yielded a sensitivity score of 80.9% (specificity = 58.2%) for the constant weighting, while for the standardized weighting, sensitivity was 80.3% (specificity = 63.0%) for the test dataset. The Receiver Operator Characteristics (ROC) curve for the scoring system using the two weighting approaches produced the same area under the curve (AUC) of 0.76, *p* < 0.001 for the test dataset. The SIREN has a good predictive accuracy of about 79% and is based on a broad range of putative risk factors associated with stroke occurrence in Africa.

#### Automated Blood Pressure Machine (Omron)

This was used to determine the systolic blood pressure of the participants in this study in mmHg.

#### Weighing Scale (Hana)

This was used to determine the weight of the participants in this study in kilograms.

#### Non-elastic measuring tape

This was used to assess the waist and hip circumferences, and was calibrated to the nearest 1 cm.

#### Height meter (locally constructed)

This was used to determine the height of the participants in this study in meters.

#### 2 ml syringes

This was used to draw 2mls of blood from the participants in this study.

#### Plain tubes

This was used to store the blood drawn from participants in this study.

#### Rolls of cotton wool

This was used to clean and pad the site of the body of the participants where blood was drawn.

#### Methylated spirit

This was used to clean the site of the body of the participants where blood was drawn.

#### Randox glucose procedure

This was used to determine the glucose level of the participants in this study in mmol/l.

#### Randox cholesterol procedure

This was used to determine the total cholesterol level of the participants in this study in mmol/l.

#### Randox 0 procedure

This was used to determine the HDL cholesterol levels of the participants in this study in mmol/l.

#### Randox LDL procedure

This was used to determine the LDL cholesterol levels of the participants in this study in mmol/l.

### Data collection

Awareness and sensitization were given to the host communities via town criers and town hall meetings to preempt of the data collection. Data was collected at meeting points like the market, major community churches, and community health centers in communities. The data collection team comprised 24 research assistants including 8 medical laboratory scientists who aided in the collection and analysis of participants’ blood samples. Participants for the research were first screened for stroke using a questionnaire for stroke-free status, and only participants with no history of stroke were selected. The following data were from the participants:

#### Bio data and risk profiles

Age, sex, occupation, educational attainment, and religion were requested verbally from the respondents. Diabetes status, smoking status, family and cardiovascular history of ailment, salt intake, vegetable consumption, sugar consumption, meat consumption, physical activity, physical stress, life events, and depression status were all verbally requested from the participants.

*Blood samples* from which the values of high-density lipoprotein, triglyceride, and blood glucose in the laboratory. Blood samples were collected from the participants in the morning by the medical laboratory scientists, centrifuged (to collect the serum), and stored in a refrigerator at 2-8ºC. The samples were then analyzed in a laboratory (at Nnamdi Azikiwe Teaching Hospital Nnewi) to determine the values of high-density lipoprotein, triglyceride and blood glucose of the participants.

#### Blood pressure

The systolic and diastolic blood pressure was measured using a standard aneroid sphygmomanometry. The participant was placed on a comfortable chair with an arm supported with a pillow-rest. He/she was then required to rest for some minutes before inflation of the cuff to avoid exaggerated values.

*Weigh*t was taken with the initial pointer at 0kg and the test subject mounted the weighing sale with their bare feet and minimal clothing and looking straight ahead. It was measured to the nearest 0.1kg.

*Body mass index and waist/hip circumference* were calculated from weight, height, hip and waist circumferences using the standard formulae.

The data collected above was then used to calculate the participants’ scores on the SIREN and MFSRS.

### Analysis of data

Data was analysed using the Statistical Package for Social Sciences (SPSS) version 22. The descriptive statistics of percentages, frequency counts, range, mean, and standard deviation were used to summarize socio-demographic variables, stroke risk factors, and the MFSRS and the SIREN scores of the participants. Kolmogorov–Smirnov’s test was used to test for the normality of the participants’ scores on the SIREN and the MFSRS. The scores were found to be skewed. Spearman-rank order correlation was then used to determine the level of correlation between the SIREN and the MFSRS to determine the validity of the MFSRS in an African environment. The correlation coefficients were interpreted as follows: *r* < 0.3 = poor correlation; 0.3–0.5 = slight correlation; 0.6–0.8 = moderate correlation, and > 0.8 = excellent correlation [37]. The alpha level was set at 0.05.

## Results

### Socio-demographic characteristics of the respondents

Three hundred and ten (310) adults (68% females; mean age = 37.2 ± 15.8 years) who were all Christians participated in this study. Eight percent of the prospective participants refused to participate in the study thereby giving a consent rate of 92%. The majority of participants were within the age of 18–34 years (51%), and attained at least a secondary (high school educational attainment (84.1%) (Table 1).

**Table 1:** Socio-demographic profiles of the participants

Variable	Class	Frequency	Percentage
Sex	Male	97	31.3
	Female	213	68.7
Age (years)	18–34	159	51.3
	35–54	104	33.5
	55–64	25	8.1
	≥ 65	22	7.1
Occupation	Unemployed	21	6.8
	Civil/private sector	23	7.4
	Trading/Business	165	53.2
	Artisan	21	6.8
	Student	68	21.9
	Others	12	3.9
Educational	None	4	1.3
Attainment	Primary	45	14.6
	Secondary	226	73.1
	Tertiary	34	11.0
Religion	Christianity	310	100

### Mean values of stroke risk and related factors

The mean percentage stroke risks among the participants estimated by the MFSRS and the SIREN were 6.79** ± **5.21 and 34.5** ± **11.9 respectively. The average age of the participants (37.2** ± **15.8 years) fell within the range of middle age. The mean body mass index (24.9** ± **5.0 kg/m^2^), systolic (124.9** ± **13.6 mmHg), and diastolic (79.3** ± **10.9 mmHg) blood pressure of the participants were all within the normal range. Participants’ mean blood concentration of triglyceride (4.9** ± **1.2 mmol/l), and total cholesterol (10.5** ± **2.2 mmol/l) were high whereas their high-density lipoprotein (1.6** ± **0.4 mmol/l) waist-hip ratio (0.9** ± **0.1) was optimal and good respectively (Table 2).

**Table 2:** Mean values of some risk factors of stroke among the participants

Variable	Range	Mean ± SD (Interpretation)
Age (years)	18–92	37.2** ± **15.8 (Middle-aged)
MFSRS total risk score (%)	0.0–22.0	6.79** ± **5.21 (minimal)
SIREN stroke risk score (%)	12.6–92.8	34.5** ± **11.9
Body mass index (kg/m^2^)	14.2–45.2	24.9** ± **5.0 (Normal)
Systolic blood pressure (mmHg)	100–180	124.9** ± **13.6 (Normal)
Diastolic blood pressure (mmHg)	58–130	79.3** ± **10.9 (Normal)
Total Cholesterol (mmol/l)	5.2–17.3	10.5** ± **2.2 (High)
Triglyceride (mmol/l)	2.3–8.6	4.9** ± **1.2 (High)
High density lipoprotein (mmol/l)	0.5–3.2	1.6** ± **0.4 (Optimal)
Low density lipoprotein (mmol/l)	1.80–6.76	4.0** ± **1.0 (Intermediate)
Waist/Hip Ratio (cm)	0.7–1.3	0.9** ± **0.1 (Good)

### Distribution of stroke risk factors among the participants

The most prevalent risk factors among the participants were hypertriglyceridemia (100.0%), meat consumption (88.1%), sugar consumption (70.6%), raised waist-hip ratio, hypercholesterolemia (45.5%), physical inactivity (43.2%), raised body mass index (42.3%), psychological stress (41.3%), hypertension (37.7%), poor income (27.4%), and failure to consume leafy vegetables (23.9%); whereas the least prevalent risk factors were poor educational attainment (1.3%), low HDL (6.8%), diabetes status (2.9%), smoking status (8.7%) and cardiovascular disease history (8.7%). The risk factors (hypertriglyceridemia, hypertension, age, smoking status, diabetes status, and low HDL) that are commonly accounted for in both the SIREN and the MFSRS occupied the first, ninth, twelfth, seventeenth, and nineteenth positions respectively in terms of prevalence. According to MFSRS, the percentage of participants with moderate/high stroke risk was 16.0% (Table 3).

**Table 3:** Distribution of participants across other different risk factors

**Risk**	**Category (f (%))**	
	**No**	**Yes**
Hypertriglyceridemia	0 (0.0)	310 (100.0)
Meat Consumption	37 (11.9)	273 (88.1)
Sugar Consumption	91 (29.4)	219 (70.6)
Raised waist-hip ratio	153 (49.4)	157 (50.6)
Hypercholesterolemia (Excessive LDL)	169 (55.5)	141 (45.5)
Physical inactivity	176 (56.8)	134 (43.2)
Overweight/obese	179 (57.7)	131 (42.3)
Psychological Stress	182 (58.7)	128 (41.3)
Hypertension	193 (62.3)	117 (37.7)
Income level, < $100 USD	225 (72.6)	85 (27.4)
Non-consumption of vegetables	236 (76.1)	74 (23.9)
Age ≥ 50 years	241 (77.7)	69 (22.3)
Family History of Cardiovascular Disease	246 (79.4)	64 (20.6)
Life Events	246 (79.4)	64 (20.6)
Sprinkling salt on food at table	247 (79.9)	63 (20.3)
Depression	261 (84.2)	49 (15.8)
Smoking status	283 (91.3)	27 (8.7)
Cardiovascular disease history	283 (91.3)	27 (8.7)
Diabetes status	301 (97.1)	9 (2.9)
Low HDL	289 (93.2)	21 (6.8)
Low educational attainment	306 (98.7)	4 (1.3)
MFSRS Moderate/high stroke risk scores	260 (84.0)	50 (16.0)

### The average weights of the stroke risk factors among the participants

The most impactful risk factors among the participants depicted as the ones with the highest average risk weights as calculated by SIREN were hypertriglyceridemia (12.0 ± 0.7), meat consumption (5.5 ± 4.0), high blood pressure (3.8 ± 9.4), low-income level (3.6 ± 2.2), non-consumption of leafy vegetable (2.4 ± 4.3), and physical inactivity (2.2 ± 2.5). The least impactful risk factors are low HDL (0.00 ± 0.00), low educational attainment (0.0 ± 0.3), diabetes status (0.3 ± 1.7), and CVD history (0.4 ± 1.4). Worthy of note may be the fact that hypertriglyceridemia, hypertension, age, diabetes status, and low HDL that are commonly accounted for in both the SIREN and the MFSRS ranked first, third, seventh, fourteenth, and sixteenth on a relative impact table respectively among the participants (Table 4).

**Table 4:** The SIREN average weight of the stroke risks among the participants arranged in descending order of magnitude

Risk factor	Mean ± SD
Hypertriglyceridemia	12.0 ± 0.7
Meat consumption	5.5 ± 4.0
High blood pressure	3.8 ± 9.4
Low-income level	3.6 ± 2.2
Non-consumption of leafy vegetables	2.4 ± 4.3
Physical inactivity	2.2 ± 2.5
Age	1.8 ± 3.3
Psychological stress	1.6 ± 2.0
Excessive waist-hip ratio	1.5 ± 2.3
Processed sugar consumption	1.4 ± 0.9
Extra salt consumption	1.0 ± 2.0
Family history of CVD	0.8 ± 1.6
CVD history	0.4 ± 1.4
Diabetes Mellitus	0.3 ± 1.7
Lo educational attainment	0.0 ± 0.3
Low HDL	0.0 ± 0.0

### Correlation between SIREN and MFSRS scores

**Fig. 1:**
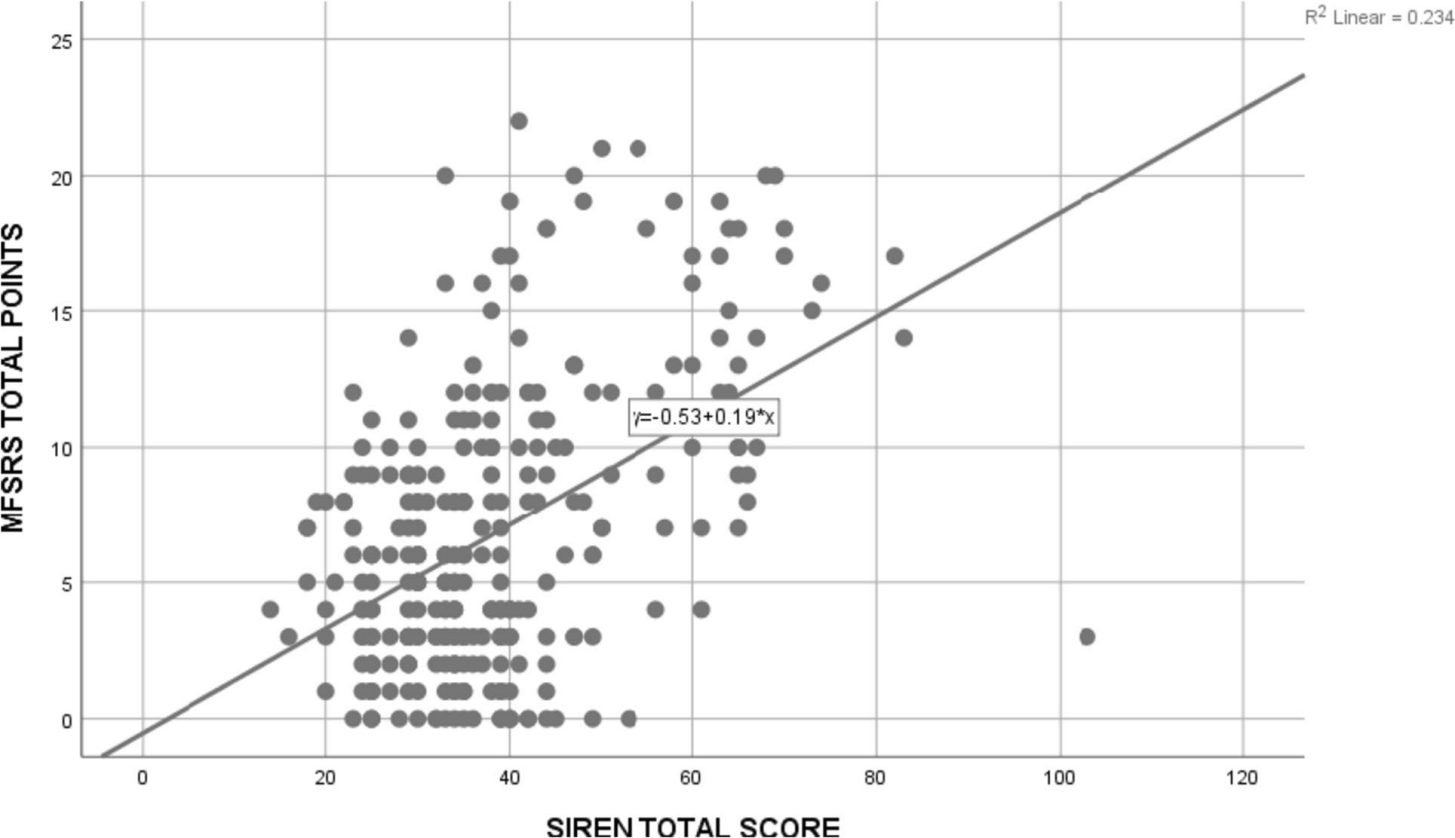
Scatter plot showing the correlation between MFSRS total score and SIREN total score

The correlation between the total scores on the MFSRS and the SIREN was weak (rho = 0.39; *p* < 0.01) indicating that the two instruments did not produce concordant results in the African environment. The correlation is pictorially represented in Fig. 1. The slope (+ 0.19) of the line of best fit on the scatter plot revealed that the two tools had a positive but not strong correlation while the intercept (-0.53) revealed that the MFSRS rated a non-significant score of -0.53 when the SIREN score was zero.

## Discussion

 This study aimed to compare the performance of the MFSRS and the SIREN in the assessment of stroke risk factors in an African (Nigerian) environment. The MFSRS was developed in a high-income country and has been a tool frequently used for the comparison of stroke risks across countries and continents [25, 26]. On the other hand, the SIREN was primarily developed for usage in low-and middle-income African countries [3]. It may be apt to compare the performances of these instruments in an African environment as this may give a better perspective and engender more understanding of stroke risk data emanating from Africa.

Present results revealed that stroke risks were highly prevalent in the present sample. This agrees with several reports about the continuous increase of stroke in African countries [6]. The most prevalent risk factors among the present participants were hypertriglyceridemia, raised waist-hip ratio, physical inactivity, psychological stress, hypertension, sugar consumption, poor income, and failure to consume leafy vegetables. Hypertension, poor diet, physical inactivity, and abdominal obesity were among the ten risk factors of stroke that accounted for 91.5% and 87.1% of the population-attributable risk for ischemic and hemorrhagic stroke in the multinational INTERSTROKE study [19]. The high prevalence of stroke risk factors in this study was further buttressed by the risk estimate of 34.5% by the SIREN. However, the MFSRS gave a low risk estimate (6.0%) and prevalence (16.0%) which is lower than stroke risks from Asian and European studies [38, 39] that had utilized the MFSRS. The MFSRS has been reported to likely underestimate stroke risks from Africa [40].

Results suggest that there may be significant differences in the rating of the SIREN and the MFSRS in the setting of this study. For example, only three out of the six stroke risks accounted for in the MFSRS made the list of the ten most prevalent risk factors in this study with the eight others being the factors only considered in the SIREN. Also, smoking status given a high priority in the MFSRS was lowly prevalent and lowly ranked risk by the SIREN in the present study while meat consumption, low income level, non-consumption of leafy vegetables, physical inactivity, and so on that were not taken cognizance of in the MFSRS were the seemingly most impactful risks in the present results according to the SIREN. Hypertriglyceridemia, hypertension, age, diabetes status, and low HDL usually accounted for in the MFSRS occupied the first, third, seventh, fourteenth, and sixteenth positions on the SIREN scale. This may be suggesting some levels of discordance in the performances of these two algorithms in the studied environment. The perceived discordance is buttressed by the presence of poor correlation between the total scores on the SIREN and the MFSRS. Some questions may thus emanate from these findings. Could the MFSRS and SIREN be respectively underestimating and overestimating stroke risks in African environments? Could there be a need to modify one or the two algorithms to more accurately assess the risks of stroke in Africa, and thus engender a more credible international comparison? Could the MFSRS justifiably have an African version that will vary from the one used in high-income countries? Consequently, there may be a yearning for further large African-centred quality control studies to ascertain this in order to improve the credibility ratings of stroke risks coming from Africa. However, the literature seems to agree with the estimates from the SIREN in the present study. The high stroke risk estimate by the SIREN in the present study is in concordance with previous reports of high risks in low-and middle-income countries [6]. On the other hand, the prevalence of stroke risks accounted for by the MFSRS in the present study was lower than the prevalence figures from higher economies [38, 39]. Furthermore, the fact that the SIREN was primarily developed for use in African countries might suggest that it would perform better than the MFSRS in the African environment.

The proportion of young adults recorded in this study reflects the pattern of Nigerian population distribution [41]. However, the educational attainment and employment rate in this study were far higher than the generality of Nigerians. This could be attributable to real improvements in the educational attainment of the population because Anambra State had been occupying a top position in education among the 36 states of Nigeria within the last 15 years [42]. The purported increase in educational attainment would understandably increase the chances of getting employed. Being a commercial centre, it may be easy to understand why the majority of the population were traders.

The present study was not without limitations. Though this was a population-based study, a larger sample size that was not restricted to a single Nigerian community might have given more power to this study. However, the fact that the two algorithms were applied among the same African population can still give some level of credence to the revealed discordance between the algorithms in an African setting. Furthermore, the quality of this study would have been improved further if some potential participants did not decline participation in the study due to erroneous fear of using their blood sample for diabolism, a usual belief in the setting of this study.

## Conclusion

Stroke risks were highly prevalent in the sampled Nigerian population with hypertriglyceridemia, raised waist-hip ratio, hypercholesterolemia, physical inactivity, psychological stress, hypertension, sugar consumption, poor income, and failure to consume leafy vegetables being the most prevalent risk factors. The poor correlation between the two algorithms and the fact that risk factors not accounted for in the MFSRS were among the most impactful according to the SIREN rating suggested some significant levels of disagreement between the two algorithms. From the literature and the global present trend of stroke risk, the MFSRS seemed to underestimate stroke risks in the present population while the SIREN seemed to agree with the literature thus suggesting that the latter might be a better predictor of stroke risks than the former in an African environment. Despite this, there is a need for large African-based quality control studies to determine the lapses (if there is any) in one or the two algorithms to improve the quality of stroke risk data emanating from Africa. Before then, there may be a need to apply caution while interpreting stroke risk estimates from Africa using these algorithms.

## Data Availability

The dataset used and/or analysed during the current study are available from the corresponding author on reasonable request.
